# l‑DOPA-Containing Protein Autoxidation: An Empirical Valence Bond Simulation of the Rate-Limiting Step

**DOI:** 10.1021/acs.jpcb.5c06223

**Published:** 2025-11-24

**Authors:** Gabriel Oanca, Alja Prah, Johan Åqvist, Janez Mavri

**Affiliations:** † Department of Cell & Molecular Biology, 8097 Uppsala University, Biomedical Center, SE-751 24 Uppsala, Sweden; ‡ Laboratory for Computational Biochemistry and Drug Design, National Institute of Chemistry, Hajdrihova 19, 1000 Ljubljana, Slovenia; § Networking Infrastructure Centre, Jožef Stefan Institute, Jamova 39, 1000 Ljubljana, Slovenia

## Abstract

Parkinson’s disease is a debilitating neurodegenerative disorder currently affecting ten million people worldwide. l-DOPA, or levodopa, is a crucial drug in addressing this issue, being a precursor of dopamine, a neurotransmitter which regulates motor functions, relieving the tremor symptom of Parkinson’s. However, l-DOPA comes with side effects that are concerning for long-term treatment. Like dopamine, which can autoxidize to dopaquinone by entering a redox cycle, l-DOPA can also be converted to dopaquinone by the same mechanism, thus becoming a continuous source of hydrogen peroxide. Furthermore, because it is structurally similar to the amino acid tyrosine, it can also get incorporated into the proteins’ sequence, thus becoming an additional source of oxidative stress for patients undergoing l-DOPA treatment. The rate-limiting step in the process of l-DOPA autoxidation is water protolysis, which yields hydroxide and hydronium ions. A similar rate-limiting step was observed in carbonic anhydrase II. In addition, the mechanism by which a hydroxide ion is transferred from bulk water was also considered. The next step, involving a nucleophilic attack by a hydroxide ion on a neutral amino group, along with cyclization, is not rate limiting. Using the Empirical Valence Bond (EVB) method, we computed the free-energy profiles for the reaction of l-DOPA incorporated into MAO A, replacing Tyr407. The calculated barrier of 33.93 kcal mol^–1^ is approximately 6 kcal mol^–1^ higher than the experimental barrier of 27.55 kcal mol^–1^ for l-DOPA in aqueous solution. The findings from our previous study of l-DOPA autoxidation in aqueous solution are critically discussed in the context of the rate-limiting step. The slow autoxidation kinetics of l-DOPA-containing proteins suggest that the main pathway through which l-DOPA induces oxidative stress is likely either the autoxidation of l-DOPA in aqueous solution or its decarboxylation, followed by dopamine autoxidation. However, a significant source of l-DOPA-induced oxidative stress may be zinc- and calcium-dependent proteins present in the central nervous system.

## Graphical abstract

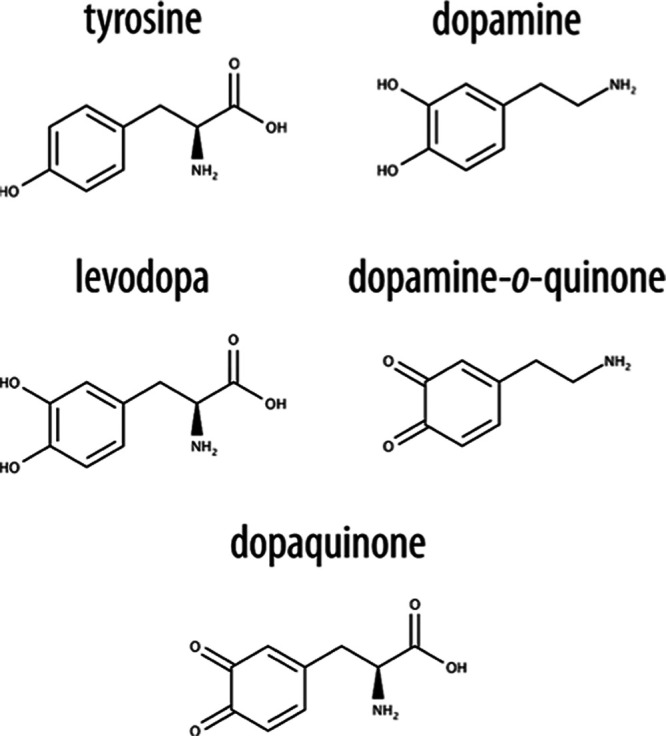

## 1 Introduction

Parkinson’s disease is a neurodegenerative disorder that affects motor skills. The drug of choice for treating Parkinson’s disease is l-DOPA, which is the short name for levodopa, acting as a precursor to dopamine and a neurotransmitter critical in motor control and other neurological functions. While other neurotransmitters, including dopamine, cannot penetrate the blood–brain barrier, l-DOPA can easily pass through. In the central nervous system, it is decarboxylated into dopamine, a reaction catalyzed by aromatic l-amino decarboxylase. To prevent its peripheral metabolism, l-DOPA is usually coadministered with the aromatic l-amino decarboxylase inhibitor, carbidopa, which cannot penetrate the blood–brain barrier.^1^ The introduction of l-DOPA in the 1960s marked a significant shift in the treatment of Parkinson’s disease, providing substantial relief from symptoms such as tremors and bradykinesia.

l-DOPA was first synthesized by Funk^2^ and later isolated from broad bean (*Vicia faba*) seeds by Gugenheim.^3^ Further work, led by Arvid Carlsson,^4^ was awarded the Nobel Prize in Medicine or Physiology in 2000, continued by Hornykiewicz,^5^ Cotzias,^6^ and Yahr^7^ who played a pivotal role in bringing l-DOPA from bench to bedside.^8^ Although l-DOPA was proved to be very effective in managing Parkinson’s symptoms, it also comes with physical and psychiatric side effects such as dyskinesias, hallucinations, and mood swings.

Structurally, l-DOPA resembles tyrosine and dopamine (Figure 1). In vivo, l-DOPA is either decarboxylated to dopamine or oxidized to its quinone form, dopaquinone. The latter transformation contributes significantly to oxidative stress through two different mechanisms: (a) autoxidation or (b) incorporation into proteins, which was only mentioned recently in the literature.^9^ Unlike dopamine which undergoes rapid autoxidation,^10^ l-DOPA has a much slower rate. Nonetheless, both dopamine and dopaquinone require the presence of a hydroxide ion in this process, producing bicyclic leukodopachrome and reactive oxygen species (ROS) as byproducts. The resulting oxidative stress of l-DOPA in shellfish proteins may also explain its strong adherence to water.^11–13^

**Figure 1:**
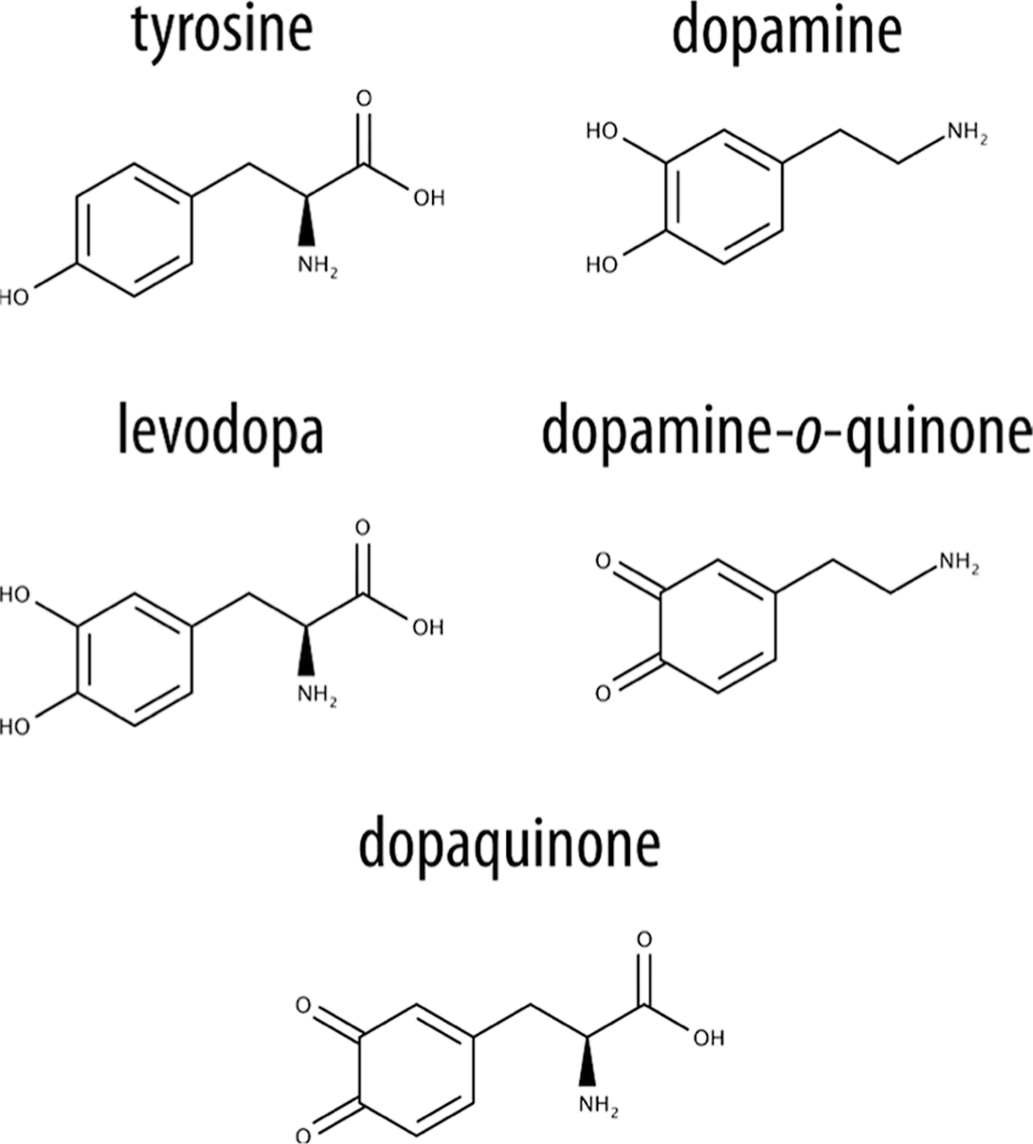
Structures of tyrosine, dopamine, levodopa (l-DOPA), dopamine-*o*-quinone, and dopaquinone.

l-DOPA is essentially tyrosine with an additional hydroxyl group vicinal to the existing one. Due to its structural similarity, ribosomes may incorporate l-DOPA during protein synthesis, replacing aromatic amino acids. An additional source of l-DOPA containing proteins is the oxidation of Tyr residues in already synthesized proteins. The latter pathway applies to all proteins and is not limited to patients with Parkinson’s disease undergoing l-DOPA therapy. It is worth emphasizing the highly stochastic nature of both (bio)­synthetic pathways, leading to the production of l-DOPA-containing proteins. In most cases, a mixture of the wild type and various mutant proteins is produced. In the presence of molecular oxygen, protein-bound l-DOPA undergoes rapid conversion to dopaquinone. This compound, like dopamine, tends to enter the redox cycle and generates hydrogen peroxide.

Prolonged exposure to ROS in the central nervous system can damage the membrane of neurons and lead to amyloid plaque formation, and both processes contribute to neurodegeneration. One way to mitigate oxidative stress is to administer l-DOPA together with antioxidants. Nonetheless, understanding the oxidative stress pathways is essential for optimizing the therapeutic benefits of l-DOPA in the treatment of Parkinson’s disease.

In our previous study on l-DOPA autoxidation, we assumed that the rate-limiting step is the nucleophilic attack of a hydroxide ion on the amino group, concerted with cyclization, in which l-DOPA has already been oxidized to its quinone form, dopaquinone.^14^ Assuming the availability of a hydroxide ion and a deprotonated amino group, this step had a barrier of 14.75 kcal mol^–1^. Furthermore, we assumed the validity of the Henderson–Hasselbalch equation for water deprotonation leading to the formation of hydroxide ions. In this work, we critically reassessed the assumptions made in our previous study. We propose water protolysis as the rate-limiting step, in accordance with the findings of previous studies on carbonic anhydrase.^15^ To this end, we employed a multiscale QM/MM methodology at the Empirical Valence Bond (EVB) level.^16^ The EVB approach, developed by Warshel in the 1980s, is the method of choice in computational enzymology because it is computationally inexpensive, allowing for reliable estimates of free-energy profiles. As a reference, we used water protolysis in bulk water, for which the reaction profile can be fully constructed from experimental data. The EVB parameters derived from this reference were then applied to compute the same reaction in monoamine oxidase A (MAO A), with l-DOPA replacing the incorporated Tyr407 residue. The energy profile for such reactions depends on the polarity of the environment, which can be water, aqueous solution of ions,^17^ or the protein matrix itself. The barrier for water protolysis in the vicinity of l-DOPA-substituted Tyr407 increased, indicating that the protein environment is anticatalytic for l-DOPA autoxidation. In conjunction with additional experimental and computational work, the results presented herein contribute to a deeper understanding of l-DOPA side effects associated with increased oxidative stress. These findings lay the foundation for the development of enhanced therapeutic strategies for the management of Parkinson’s disease.

## 2 Methodology

### 2.1 Experimental Kinetic Data of l-DOPA Autoxidation

Experimental l-DOPA autoxidation kinetics in aqueous solution was studied at 37 °C and pH 7.4.^18^ The rate-limiting step is dopaquinone cyclization, with a rate constant of 2.56 × 10^–7^ s^–1^, corresponding to a half-life of 752 h. Using the Eyring–Polanyi equation, this rate constant results in an activation free energy of 27.55 kcal·mol^–1^.

### 2.2 Water Protolysis: Experimental Facts

Water protolysis is a slow reaction, formally defined as the formation of a hydroxide ion and a hydronium ion at a separation corresponding to a 10^–7^ mol·L^–1^ concentration, from two water molecules, forming a contact hydrogen bond:

1 $$2H_{2}O⇌H_{3}O^{+}+OH^{-}$$

For bulk water, experimental data are available, while for other environments, the experimental data are limited. For bulk water at 25 °C and pH 7, the concentrations of both H_3_O^+^ and OH^–^ are 1 × 10^–7^ mol·L^–1^, and the concentration of H_2_O is 55.345 mol·L^–1^. With these values, it is possible to calculate equilibrium constant *K*_eq_, which has a one-to-one correspondence with the reaction free energy. Contrary to the assertions made in several textbooks, a true equilibrium constant must be dimensionless, and all concentrations must be expressed in terms of mole fractions. In our case, the expression for the equilibrium constant becomes

$$K_{eq}=\left(\frac{1{\cdot 10}^{-7}}{55.345}\right)\times \left(\frac{1{\cdot 10}^{-7}}{55.345}\right)/1={\left(\frac{1{\cdot 10}^{-7}}{55.345}\right)}^{2}$$ 2

where the mole fraction for water is unity, hence 1 in the denominator. This expression yields a dimensionless value for the equilibrium constant from which the free energy can be calculated, and it is equivalent to using a 55.345 M standard state. The calculated value for *K*_eq_ is 3.25 × 10^–18^, corresponding to the reaction free energy Δ*G*_0_ = 23.88 kcal·mol^–1^ calculated as

3 $$\Delta G_{0}=-k_{B}T\ln\left(K_{eq}\right)$$

Δ*G*_0_ value for this process can also be calculated from the p*K*_a_ values of −1.7 and 15.7 for hydronium and hydroxide ion, respectively, using the Henderson–Hasselbalch equation:

4 $$\Delta G_{0}=1.38\times \left(15.7+1.7\right)=24.0\;{kcal\cdot mol}^{-1}$$

The good agreement between the calculated Δ*G*_0_ values for water protolysis using these two methods confirms that the established p*K*_a_ values for water are correct. The novel suggested water p*K*_a_ values of 0 and 14 ^19^ yield a Δ*G*_0_ value of 19.32 kcal·mol^–1^, which clearly underestimates the reaction free-energy value calculated from mole fractions. The rate constant for the reverse reaction, i.e., the recombination of hydronium and hydroxide ions, was measured by Eigen and DeMayer^20^ using ultrafast electric discharge, and the obtained value was 1.3 × 10^11^ s^–1^. This corresponds to the Grotthuss mechanism of proton transfer for recombination of H_3_O^+^ and OH^–^ that are separated by at least one water molecule. The barrier for the reverse reaction can be calculated using the Eyring–Polanyi equation which yields a value of 2.33 kcal·mol^–1^. Accordingly, the forward barrier is the sum of the reaction free energy, 23.88 kcal·mol^–1^, and the reverse reaction barrier, 2.33 kcal·mol^–1^, the total calculated value thus becoming 26.21 kcal·mol^–1^. Eigen and DeMaeyer’s measurements did not extend to the final step of the recombination process, i.e., the formation of two water molecules from a contact ion pair, H_3_O^+^ and OH^–^, which consists of a highly exergonic proton transfer. They estimated that this rate constant ranges from 10^11^ s^–1^ to 10^14^ s^–1^. Proton transfer for this reaction was simulated by a mixed quantum/classical simulation, using the Density Matrix Evolution method, and the obtained value for the rate constant was 1.8 × 10^13^ s^–1^, corresponding to a barrier of 0.59 kcal·mol^–1^ (A. van der Vaart, J. Mavri, and H.J.C. Berendsen, unpublished). It should be emphasized that this small barrier has a negligible effect on the kinetics of water protolysis. The complete reaction profile for water protolysis is shown in Figure 2.

**Figure 2:**
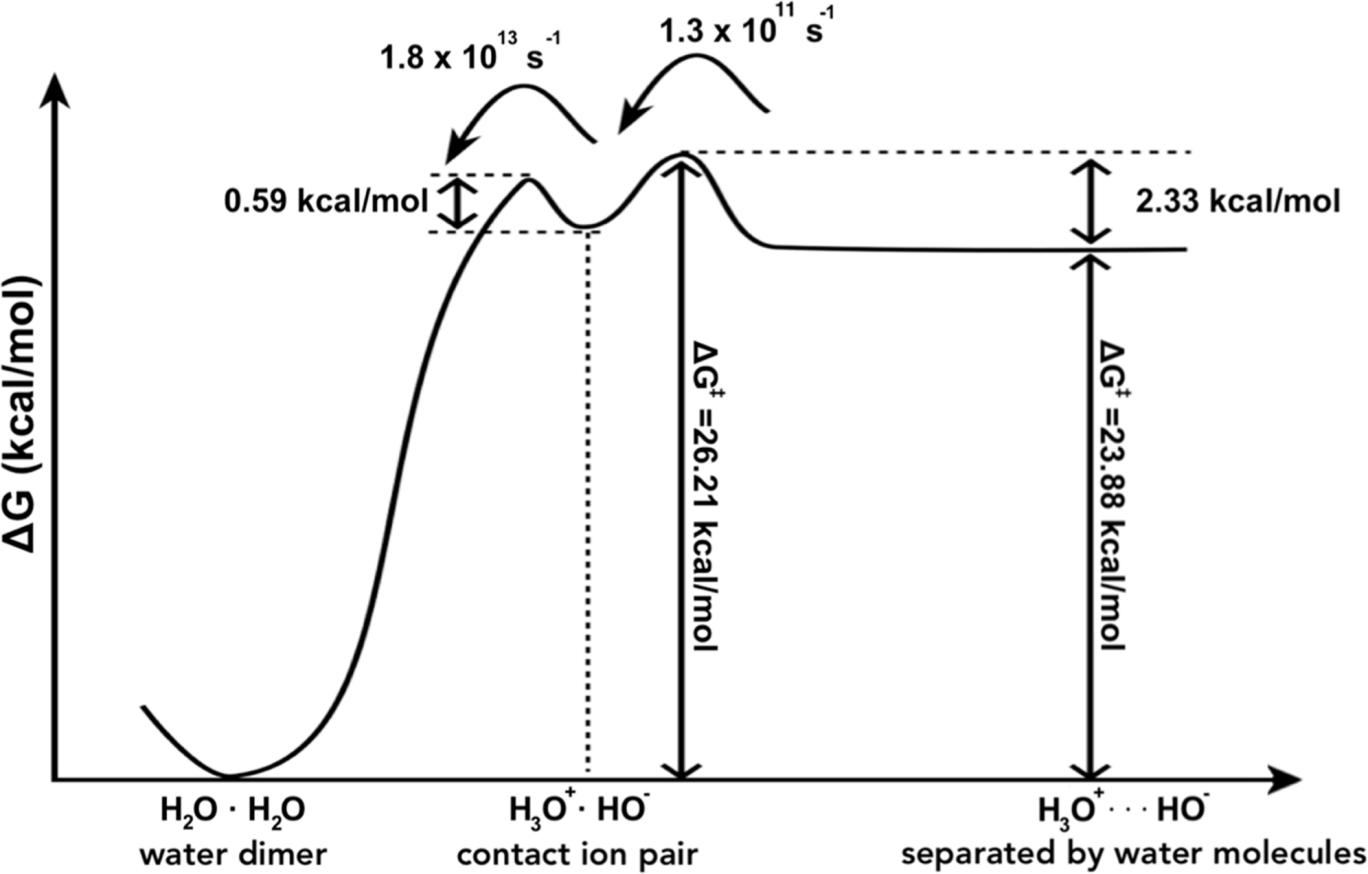
Reaction profile for water protolysis in bulk water. The reactant minimum corresponds to a hydrogen-bonded dimer. The first transition state is proton transfer giving rise to the second minimum corresponding to contact ion pair H_3_O^+^ and OH^–^. The transition state that follows is for proton transfer Grotthuss mechanism that controls the overall process. Plateau of the products H_3_O^+^ and OH^–^ is separated by one or more water molecules. Activation free energy, Δ*G* ^⧧^, is therefore 26.21 kcal·mol^–1^ and reaction free energy, Δ*G*_0_, is 23.88 kcal·mol^–1^.

Since the Grotthuss mechanism involving several reactive water molecules is not a very practical reference reaction, we have instead postulated the recombination of the contact ion pair, H_3_O^+^ and OH^–^, as a reference reaction. This reaction profile then corresponds to the Grotthuss mechanism, with a barrier of 26.21 kcal·mol^–1^ and a reaction free energy of 23.88 kcal·mol^–1^. In this regard, the complexity of Grotthuss mechanism is absorbed into the parametrization of water dimer protolysis where both resulting ions form an ion pair. The same approach was applied in other studies involving water protolysis.^15^

### 2.3 Critical Comments Concerning Our Previous Work on l-DOPA Autoxidation

In our previous work,^14^ we modeled a two-step reactive profile of l-DOPA autoxidation in water. First, we simulated the reaction profile with the hydroxide ion in bulk water, yielding a barrier of 18.13 ± 1.12 kcal·mol^–1^. The validity of the Henderson–Hasselbalch equation was assumed for the creation of a hydroxide ion from a water molecule at pH 7.4. Reversible work for this step is Δ*G* ^OH–^ = *k*_B_ *T* ln(10)·(*pK*_a_(OH^–^) – pH) = 1.38·(15.7–7.4) = 11.79 kcal·mol^–1^. The summation of these two values results in a barrier of 29.92 kcal·mol^–1^. It is also necessary to add a small contribution from amino group deprotonation, Δ*G* ^NH_2_^ = *k*_B_ *T* ln(10)·(p*K*_a_NH_2_ – pH) = 1.38·(8.11–7.4) = 1.01 kcal·mol^–1^, yielding the activation energy of 30.93 ± 1.12 kcal·mol^–1^. The agreement with the experimental barrier of 27.55 kcal·mol^–1^ is far from being very good, but it is nevertheless acceptable. Our previous approach is however problematic because water protolysis is an extremely rare event, which for a tagged water dimer takes place only once in 270 days, and the Henderson–Hasselbalch equation is not valid for this process. The protolysis event must take place in close proximity to the potential site for the reaction, so the resulting hydroxide ion can immediately enter the reaction with l-DOPA, which has a much lower barrier and is therefore not rate limiting. Hence, the correct rate-limiting step for l-DOPA autoxidation is water protolysis in the immediate vicinity of l-DOPA. A slightly elevated barrier relative to bulk water is anticipated, as l-DOPA partially shields the water dimer and the resulting ions. Therefore, the experimental barrier of 27.55 kcal·mol^–1^ is reasonably comparable to the barrier for water protolysis of 26.21 kcal·mol^–1^. An important finding from our previous study is that the barrier for the nucleophilic attack of a hydroxide ion on l-DOPA, concurrent with cyclization, is 18.13 ± 1.12 kcal·mol^–1^, which is unequivocally not rate limiting. Therefore, the rate-limiting step is water protolysis, and the same assumptions and computational approaches employed in the analysis of rate-limiting steps of carbonic anhydrase I and neutral ester bond hydrolysis apply to this reaction.^15,21^

### 2.4 System Preparation and Force Field Parametrization for the Tyr407L-DOPA Mutant of Monoamine Oxidase A

The structure for l-DOPA-mutated MAO A was built based on the crystal structure with PDB ID 2Z5X from Protein Data Bank^22^ and flavin-adenine-dinucleotide cofactor (FAD) was bonded to the Cys406 residue. The substitution of Tyr407 to l-DOPA was performed using UCSF Chimera software.^23^ The potential targets for l-DOPA mutations are Tyr197, Tyr407, and Tyr444, which form the reaction site. Among these three, Tyr407 was identified as the sole residue to which water can penetrate its amino group, with one crystallographic water (WAT 787) being already present at 3 Å distance. Moreover, after solvating and equilibrating the system, additional water molecules penetrated the proximity, forming two potential reactive water pairs and a continuous water chain extending from the enzyme to the exterior, which are suitable for facilitating a Grotthuss mechanism. The equilibrated reaction site in reactant configuration is shown in Figure 3.

**Figure 3:**
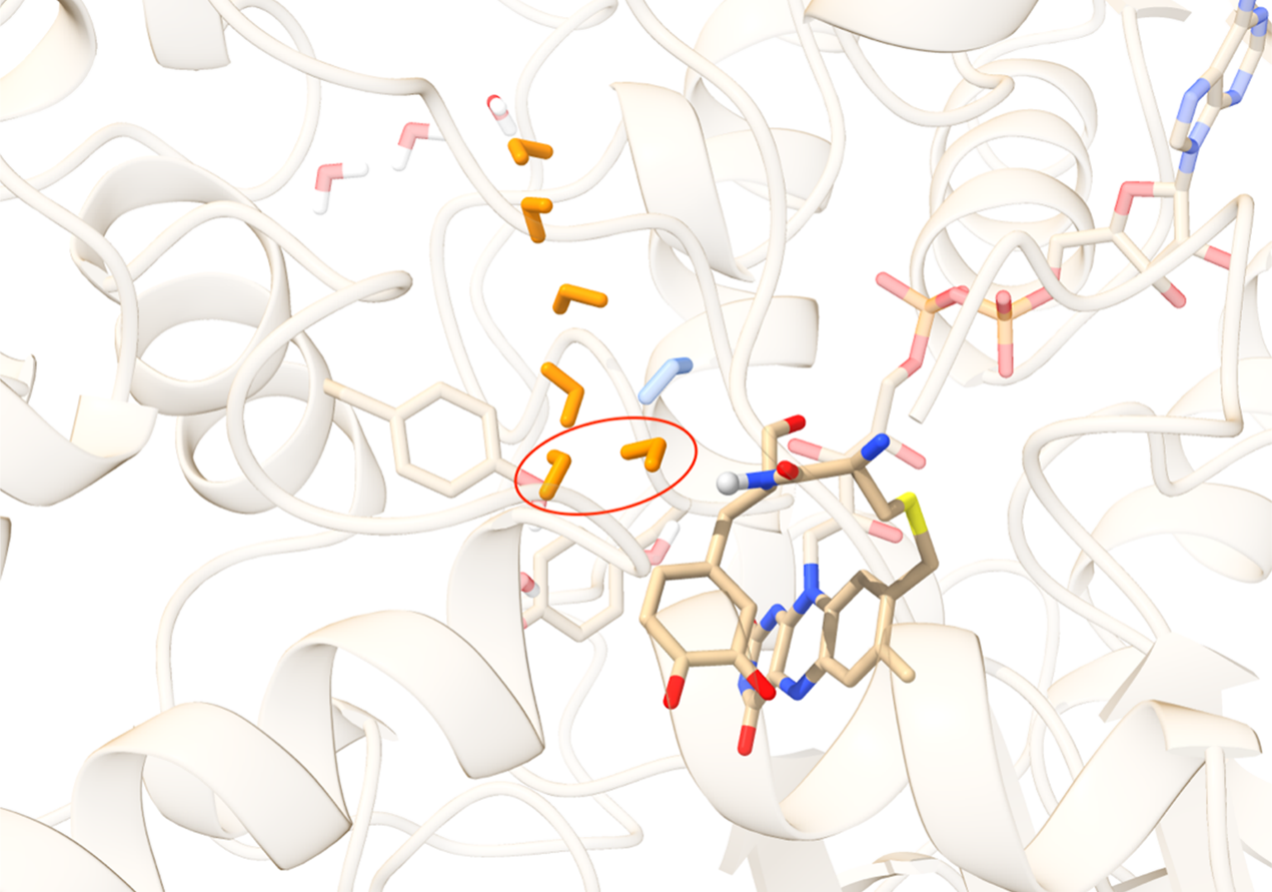
Initial structure for Tyr407L-DOPA MAO A mutant active site, with l-DOPA in its quinone form. The reactive amino hydrogen is shown as a gray sphere, the water chain is depicted in orange, the second potential proton acceptor molecule is shown in blue, and the reactive water molecules are encircled in red.

OPLS-AA force field^24^ was used to model the enzyme. For the FAD cofactor, which is not included in the standard force field, we applied the parameters from our previous work.^25^ In that study, restrained electrostatic potential (RESP) charges^26^ were derived using AmberTools,^27,28^ based on electrostatic potential (ESP) charges generated with Gaussian09,^29^ following geometry optimization at the HF/6-31G­(d) level of theory. van der Waals and bonded parameters were obtained using the ffld_server tool from Schrödinger.^30^

The same procedure was employed to generate the force field parameters for l-DOPA in its quinone form. Several conformations of the mutated residue were evaluated, and the conformer exhibiting minimal steric strain, as shown in Figure 3, was selected. The protonation states of MAO A residues were consistent with our previous studies.^14^ The new enzymatic system was solvated in a spherical water cell with a radius of 30 Å centered on the N5 atom of lumiflavin, as in our earlier work. This setup included 1884 water molecules, represented by the TIP3P water model.^31^

The Empirical Valence Bond (EVB) method^16^ considers two states: the reactant state (RS) and the product state (PS). In our case, the RS is represented by a hydrogen-bonded water dimer, while the PS corresponds to a hydronium–hydroxide ion pair. We considered two pairs of reactive water molecules, shown in Figure 3, in which the crystallographic water molecule located near the amino group of the mutated Tyr407L-DOPA acts as a proton donor, being transformed to a hydroxide ion in the product state. In addition to testing two different pairs of reactive waters, we also evaluated several parametrizations. The tested parameters and their corresponding reaction profiles are provided in the Supporting Information file (Figures S1–S5 and Tables S1–S5). We initially employed TIP3P parameters (charges, van der Waals, and angle terms) for H_2_O and the ffld_server tool generated parameters for hydronium and hydroxide ions. The harmonic bonds were replaced with Morse potentials using various parameter sets as described in the Supporting Information. After multiple trials, we selected the set of charges and Morse parameters used in a previous study of water protolysis near ions,^17^ summarized in Table 1.

**Table 1:** Applied EVB Parameters for Water Protolysis^e^

		Lennard-Jones^b^		Buckingham repulsion^c^	
atom types	atomic charges^a^	A	B	C	β
ohh.O	–0.80	762.8900	24.3900	20.0	1.58
ohh.H	0.40	6.2400	1.4700	50.0	1.58
wpp.O	–0.08	762.8900	24.3900	--	--
wpp.H	0.36	6.2400	1.4700	50.0	1.58
oh-.O	–1.01	976.9297	31.2559	20.0	1.58
oh-.H	0.01	69.5797	4.9095	--	--
Na+	1.00	143.6955	3.8899	--	--

Morse bonds^d^			
Atom pair	D_e_	β	r_0_
O–H	109.1	2.0	1.0

EVB parameters		energies	water	enzyme
H_ij_	66.85	Δ*G* ^⧧^	26.21 ± 0.09	33.93 ± 0.96
gas-shift	209.70	Δ*G*_0_	23.88 ± 0.20	--

a Atomic charges from ref 17.

b Lennard-Jones (LJ) parameters for the reactive oxygen atoms in the reactant state were taken from the TIP3P model. For all hydroxide atoms and for hydronium oxygen atom, we used LJ parameters from ffld_server, while for hydrogen atoms of water and hydronium, the LJ parameters were calculated as described in the Supporting Information.

c The Buckingham-type potential has been applied for atom pairs involved in breaking and forming bonds.

d Morse potential was applied to all EVB bonds.

e Lennard-Jones parameters are provided in the Q5 program format, i.e., as the square root of their standard form, due to the use of the geometric combination rule. wpp stands for hydronium residue type. Please note that the reaction profile in enzyme does not exhibit a distinct minimum for the products, but only a shoulder on an ascending slope, which we identified as the transition state. All values are given in AKMA units.

Small Lennard-Jones centers were added to all hydrogen atoms, and all O–H bonds were modeled using Morse potentials. In standard force fields, Lennard-Jones parameters for most hydrogen atoms are typically set to zero as these atoms are usually located within the van der Waals (vdW) radius of their bonded heavy atom. However, this assumption is invalid during proton transfer, where hydrogen atoms can become exposed. Additionally, the weaker Morse bonds compared to their TIP3P harmonic counterparts were insufficient to counteract the Coulomb interactions with nearby water. This behavior was observed not only for the hydrogen reactive bond but also for all Morse bonds in the EVB region. To address this, Lennard-Jones parameters for these hydrogens were added so that their vdW spheres, when interacting with oxygen atoms from the first solvation shell, would match the typical oxygen—oxygen vdW distance minus the oxygen–hydrogen equilibrium bond length. We also replaced the Lennard-Jones interactions between atom pairs directly involved in bond formation and cleavage during the reaction process with Buckingham-type potentials of the form *C*·*e* ^–βr^. The two reactive waters were also restrained by flat-bottom harmonic potentials at 2.5 and 3.5 Å from each other, using a 10 kcal·mol^–1^·Å^–2^ harmonic potential above and below those distances. All simulations and free-energy calculations were carried out using the Q5 software package.^32^

### 2.5 Computational Details

The system was first equilibrated, starting with harmonic positional restraints on the entire system. During this process, the temperature was gradually increased from 1 to 300 K while progressively releasing the restraints, except for 0.5 kcal·mol^–1^·Å^–2^ maintained on the EVB heavy atoms (*i.e.*, the two reactive oxygen). The integration time step was set to 1 fs, except during the initial 50 ps of equilibration, where a shorter time step of 0.1 fs was used. Following this initial setup, all subsequent simulations were performed at 300 K with a time step of 1 fs. A spherical cutoff of 10 Å was applied to protein–protein, protein–water, and water–water interactions. For long-range interactions beyond this distance, the local reaction field (LRF) method^33^ was employed. No cutoff was applied to the EVB region, for which pairwise interactions were calculated for the entire system.

After equilibration, we performed a short Free-Energy Perturbation (FEP) simulation, gradually transforming the system from reactant to product configuration over 51 FEP windows (Δλ = 0.02), each 10 ps in duration. From this run, we selected the configuration corresponding to the transition state (TS), typically located at λ = 0.5, and further equilibrated it for 1 ns. This equilibrated TS configuration was then used as a starting point for the production FEP simulation. In the production phase, the FEP transformation was carried out in both directions, from the TS toward the reactant (RS) and product (PS) states, in steps of Δλ = 0.02, each window being simulated for 100 ps, totaling 5.1 ns of molecular dynamics. Initiating the FEP from the TS configuration helps to minimize bias toward either reactant or product low-energy states. This entire procedure was repeated 10 times to ensure statistical convergence, and each replica began with a 100 ps equilibration phase initialized with randomized atomic velocities to assess statistical independence.

We assumed that the rate-limiting water protolysis occurs in the vicinity of reactive l-DOPA inside the enzyme. An alternative scenario is that the hydroxide ion is transferred from bulk water to the vicinity of l-DOPA. Reaction free energy for this process is a sum of free energies for hydroxide ion formation in bulk water and hydroxide ion solvation in protein minus hydroxide ion hydration free energy. It should be stressed that this is the lowest limit for activation free energy, since additional barriers are anticipated during the transport.

Hydroxide ion hydration at the reactive location inside the MAO A enzyme was performed through several thermodynamic cycles using GROMACS 2022 package^34,35^ and OPLS-AA/M force field.^36^ The same ffld_server parameters for lumiflavin (FAD) and l-DOPA from the EVB simulations were added to GROMACS’ force field using gmxtools.^37^ The reactive water in the vicinity of the l-DOPA amino group was selected for the alchemical transformation. In these calculations, only charges were transformed while preserving van der Waals interactions. We used the same parameters for H_2_O and OH^–^ from the EVB simulations, except for bonds, whose parameters were taken from the TIP3P model and ffld_server for H_2_O and OH^–^, respectively. In conclusion, the only difference in parametrization consisted of using standard harmonic bonds instead of Morse for the alchemical moieties. The hydration energies for systems containing OH^–^ were evaluated in several steps, following the protocol from ref 38. The enzymatic system was encapsulated in a triclinic box with edges placed at 15 Å from the protein’s surface, solvated by 35750 water molecules represented by the SPC/E model.^39^ The system for bulk water simulations consisted of a dodecahedron with edges at 30 Å distance from the solute, solvated by 5238 SPC/E water molecules. The water and enzyme systems containing OH^–^ were neutralized by replacing one water molecule with a sodium counterion.

The MAO A system was equilibrated by starting with minimization on the steepest descendent slope. Then, we slowly heated the system from 5 to 300 K and released the positional restraints from all protein’s heavy atoms—including the counterion for systems containing OH^–^—from 200 to 0.5 kcal·mol·A^–2^ in nine consecutive steps of 100 ps each, at 1 fs step-size. The first five steps were performed in an *NVT* ensemble with an isotropic V-rescale thermostat. The next four steps were performed in an *NPT* ensemble with an isotropic C-rescale barostat. After these nine steps, we continued for another 1 ns equilibration and 1 fs integration time step, with 0.5 kcal·mol^–1^·Å^–2^ restraints on all protein’s heavy atoms, followed by 100 ns of molecular dynamics (MD), at 2 fs time step and no positional restraints.

In the enzyme, the reactive waters were restrained at the reaction site using a flat-bottom harmonic potential between 2.5 and 3.5 Å from the N atom of the l-DOPA amino group and 10 kcal·mol^–1^·Å^–2^ otherwise. The counterion was also restrained using a flat-bottom harmonic potential between 42.0 and 44.0 Å from the Cα atoms of residues Asn170 and Cys395, and 10 kcal·mol^–1^·Å^–2^ otherwise. The resulting confining space in which the counterion was free to move is shown in Figure 4.

**Figure 4:**
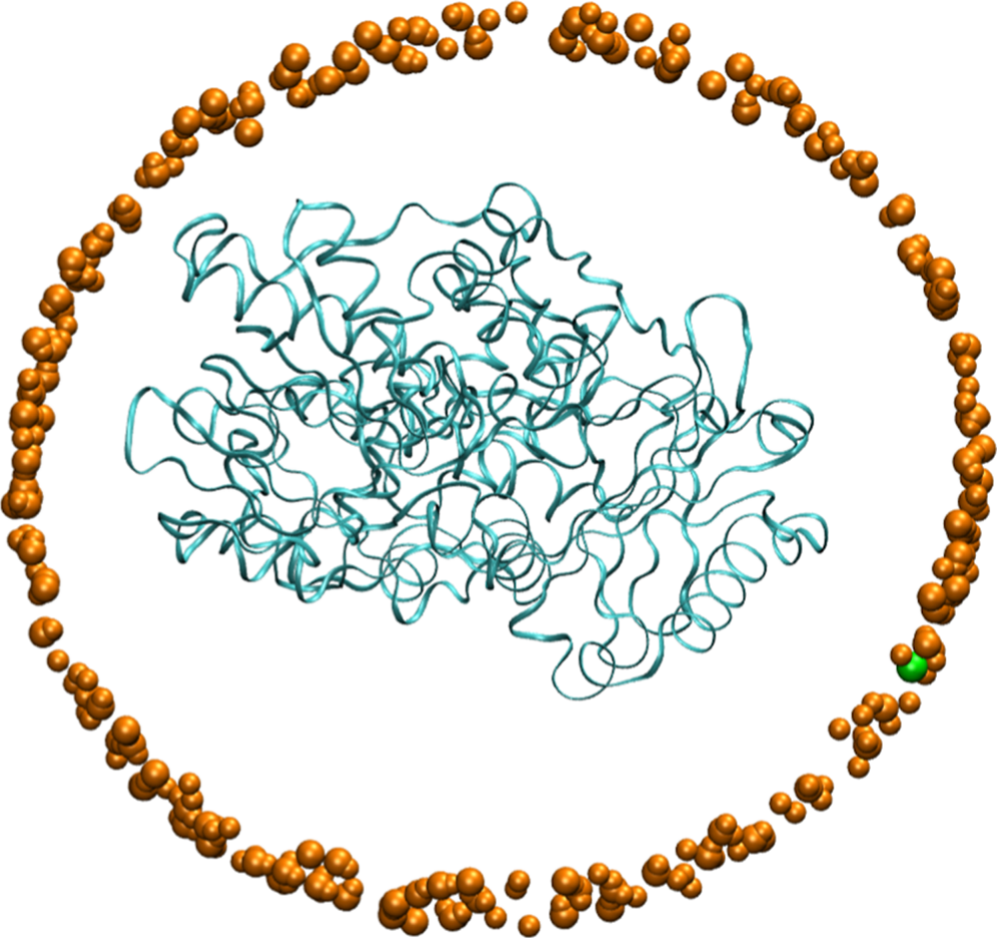
MAO A enzymatic system containing OH^–^ and one sodium counterion. The enzyme is shown in blue ribbon and the counterion is shown as a green sphere. The orange spheres are solvent molecules shown for the purpose of indicating the confining space for the counterion.

The system for bulk water was equilibrated similarly as the enzyme, except that the first nine relaxation steps consisted of only 20 ps each, and the 10th step was only 100 ps long, all with 20 kcal·mol^–1^·Å^–2^ harmonic restraints on the reactive heavy atom at the center of the solvation box. This was followed by two additional nanoseconds MD at a 2 fs time step and 10 kcal·mol^–1^·Å^–2^ harmonic restraints. For the water system containing OH^–^, the counterion was also restrained by a flat-bottom harmonic potential between 20.0 and 24.0 Å from the reactive oxygen, and 10 kcal·mol^–1^·Å^–2^ otherwise.

The equilibration was followed by the production run, where the alchemical transformation was performed in 21 FEP windows, linearly transforming the charges in incremental steps of 0.05. At each FEP window, we performed three MD simulations, each 1 ns long and with a 1 fs integration time step: (i) *NVT* ensemble simulation using a V-rescale thermostat and 100 fs for temperature coupling; (ii) *NPT* ensemble simulation adding a C-rescale barostat with 1 ps pressure coupling; (iii) production run for data collection, with the same setting from the previous *NPT* step, except for pressure, which was coupled every 100 ps. Data was collected every 100th step and analyzed by the Bennett Acceptance Ratio^40^ using the BAR tool from GROMACS package. All thermodynamic cycles were performed in batches of 5 replicas, each starting with a 1 ns MD equilibration and random initial velocities to assess statistical convergence. For thermodynamic cycles starting from a different initial state than the equilibrated one, we performed additional equilibrations, 10 ns for enzymatic systems and 1 ns for water systems, with a 2 fs time step.

## 3 Results and Discussion

The molecular dynamics (MD) trajectories for the rate-limiting step were generated using the mapping potential from eq 5, where the driving potential is gradually transformed from reactants into products through a coupling parameter λ:^41^

5 $$V\left(\lambda \right)=\lambda \varepsilon_{1}+\left(1-\lambda \right)\varepsilon_{2}$$

where ε_1_ and ε_2_ represent the potential energies generated with RS and PS force fields, respectively. The associated free-energy profiles were computed using the well-established Free-Energy Perturbation/Umbrella Sampling approach.^42–44^

In addition to simulations in the enzymatic environment, the same reaction was also carried out in an aqueous solution. The free-energy profile in water was fitted to the experimental barrier height of 26.21 kcal·mol^–1^ and a reaction free energy of 23.88 kcal·mol^–1^. This mapping yielded calibrated EVB parameters: the off-diagonal coupling element *H*_*ij*_ = 66.85 kcal·mol^–1^ and the gas-phase shift α = 209.70 kcal·mol^–1^. These calibrated parameters were then applied to the enzymatic reaction, allowing for a direct comparison of the reaction energetics between the two environments. The resulting free-energy profiles for the reaction in water and in the mutated Tyr407 MAO A enzyme are shown in Figure 5.

**Figure 5:**
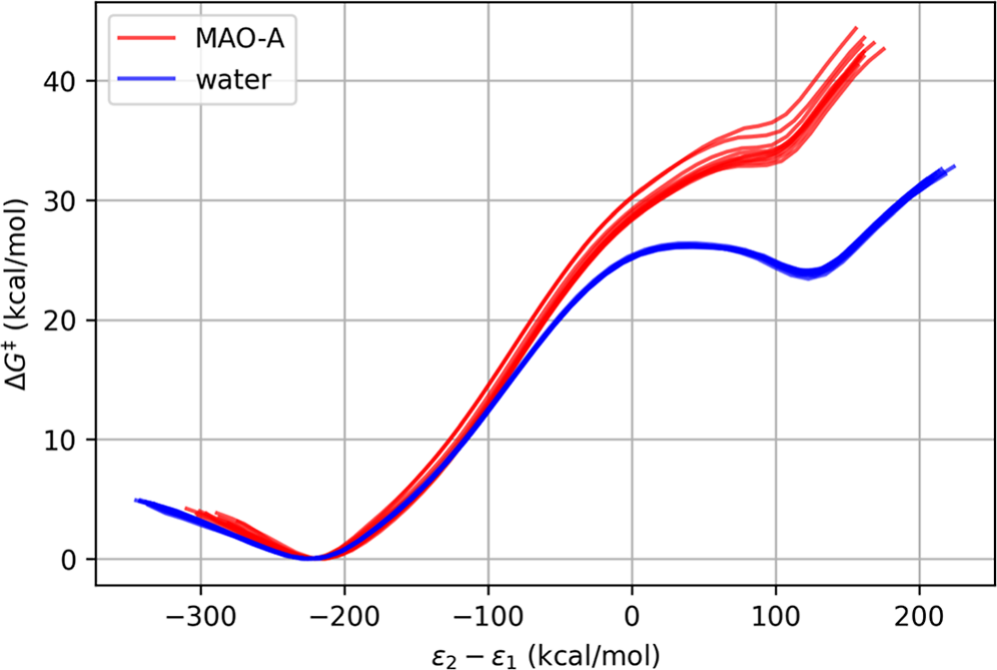
Reaction profiles for water protolysis. The profiles in the aqueous phase are shown in blue, while those for the l-DOPA-mutated MAO A are shown in red. The reaction coordinate is defined as the energy difference between the two EVB valence states, as commonly used for presenting free-energy profiles.

Figure 5 clearly shows that the MAO A environment is less favorable for water protolysis in comparison to the aqueous environment. The enzymatic profile displays only a shoulder rather than a distinct minimum for the products. The point of lowest slope on the shoulder was identified as the transition state, corresponding to an energy of 33.93 kcal·mol^–1^. In this regard, the MAO A environment is approximately 6 kcal·mol^–1^ less catalytic than the aqueous solution. This provides substantial evidence that l-DOPA incorporated into MAO A does not serve as the predominant reaction channel for its autoxidation and the associated oxidative stress.

The reported thermodynamic values correspond to the encircled pair of water molecules shown in Figure 3. During the equilibration phase, an additional water molecule entered the vicinity, which may serve as a potential proton acceptor (shown in blue in Figure 3). The reaction barrier for this pair is slightly higher, 35.07 kcal·mol^–1^, and it does not form a water chain leading out of the enzyme. The corresponding reaction profiles for this second pair are provided in Figure S6 of the Supporting Information file.

Water protolysis is a thermodynamically demanding process, and to catalyze this reaction, evolution has employed metal ions such as zinc in carbonic anhydrase II,^15^ alongside a preorganized nonmetallic enzymatic environment. In principle, the reaction in MAO A could be accelerated by transporting metal ions from the aqueous solution to the active site. However, the thermodynamic cost of this process would most likely exceed the catalytic gain achieved by decreasing the activation barrier.

Another mechanism for effective catalysis would require a water chain connecting l-DOPA to the bulk solvent, facilitating proton transfer via the Grotthuss mechanism. The studied mutant has already formed a chain of hydrogen-bonded water molecules connecting l-DOPA to bulk water; therefore, no additional energetic cost is required for its formation. Moreover, in MAO enzymes, the entry of monoamine substrates into the active site is consistently accompanied by a few water molecules, as demonstrated by molecular simulations.^14,45^ This makes the transport of water molecules to the reactive site of MAO A relatively inexpensive, in terms of free energy. For other enzymes, it is necessary to verify through molecular simulations whether access of water to l-DOPA is thermodynamically favorable. If it is not, the reaction barrier must be adjusted by adding the reversible work required to transfer two water molecules from bulk water to the reaction site, an expensive approach as it requires evaluating the cage effect for the reference reactions in water, where the formation of the reaction complex is not always thermodynamically favorable. For further discussion, see: refs 46–48 .

Evaluating binding or solvation free energies is usually performed through alchemical transformations that involve the creation or annihilation of intermolecular interactions, generally in small increments via FEP/MD simulations. Making use of appropriate thermodynamic cycles, one can assess the free energies of interest with high accuracy.

Computing solvation or binding free energies with MD simulations is more difficult though for species carrying a net charge than for neutral molecules as one must account for the missing interactions at the liquid–vacuum interface of the simulated system. When solvation free energies via thermodynamic cycles are compared, if the net charge of a system does not change with the alchemical transformation, the missing interactions would usually cancel out. But if the net charge of the system is not conserved, then one must analytically account for the missing contribution. For simulations with a spherical boundary condition, we apply a Born correction^49^ which, in its simplest form, becomes

6 $$\Delta G_{Born}=-\frac{166}{R}\left(\frac{\epsilon -1}{\epsilon }\right)\left(q_{B}^{2}-q_{A}^{2}\right)$$

where *q*_B_ and *q*_A_ are the system’s charge in its final and initial state, respectively, R is the distance from a moiety with charge *q* to the liquid–vacuum interface, and ϵ is the dielectric constant beyond the distance *R*, which is often the dielectric constant of water. This approach is formally correct for small, charged moieties, like ions, which can be easily restrained at the center of the simulation sphere, but it becomes challenging for alchemical transformations of large molecules or for heavily charged systems.

In MD simulations that use periodic images with Ewald lattice summation and tinfoil boundary condition,^50^ one must neutralize the net charge of the system, usually by adding counterions, or the long-range electrostatic potential will be affected by its periodic images.^51^ As above, this effect cancels out in alchemical transformations, where the net charge does not change between the end states. Otherwise, the free energy will be shifted by the amount given in eq 7:^50,52^

7 $$\Delta U=\frac{k_{e}\xi }{2L\epsilon }\sum_{i}q_{i}^{2}$$

If the system’s net charge differs between the end states of the simulation, then the following correction is needed:

8 $$\Delta U=\frac{k_{e}\xi }{2L\epsilon }\left(q_{B}^{2}-q_{A}^{2}\right)$$

where *q*_B_, *q*_A_, and ϵ are the same as in eq 6, *k*_e_ is the Coulomb constant, *L* is the length of the periodic solvation box, and ξ is the missing self-energy term from the periodic images that evaluates to −2.8374.^53^

In this study, we performed the hydration free energy of a hydroxide ion and a water molecule in GROMACS, using particle mesh Ewald for the long-range electrostatics with tinfoil, following the protocol from ref 38. This protocol requires several thermodynamic cycles and the use of a counterion for the self-energy term to cancel out. The alchemical transformations for the charged system are given in eqs 9 to 12, while water hydration requires only one thermodynamic cycle. More details about the thermodynamic cycle method are provided in Supporting Information.

9 $${OH}^{0}+{Na}^{0}\to {OH}^{-}+{Na}^{+}$$

10 $${{OH}^{0}+{Na}^{+}\to OH}^{-}+{Na}^{0}$$

11 $${OH}^{0}\to {OH}^{-}$$

12 $${Na}^{0}\to {Na}^{+}$$

where OH^0^ and Na^0^ are the discharged hydroxide and counterion, respectively.

The self-energies from eq 8 cancel out in cycle 10, and the solvation free energy for the hydroxide ion can be described as

13 $$\Delta G_{{OH}^{-}}=\frac{\Delta G_{9}+\Delta G_{10}}{2}$$

where Δ*G*_9_ and Δ*G*_10_ are the solvation free energies from eqs 9 and 10, respectively. The free energy for sodium can also be obtained as

14 $$\Delta G_{{Na}^{+}}=\frac{\Delta G_{9}-\Delta G_{10}}{2}$$

Hydration free energies in bulk water and in enzyme corresponding to cycles from eqs 9–12 and those for H_2_O are reported in Table 2. Having a counterion alongside the hydroxide in the same box can result in a mutual electrostatic interaction between the two which, just as the self-energy, cancels out in eq 10. In water, for an average distance of 22 Å between ions, the analytical estimation amounts to 0.1 kcal·mol^–1^. From it, we can also assess the self-contribution, which is 0.03 kcal·mol^–1^. We expect that these values are even smaller in the enzyme, given the bigger box, so we will ignore them.

**Table 2:** Hydration Free Energies for H_2_O and OH^–^ ^a^

solute	step	water	MAO A
H_2_O	Δ*G*_H2O_	–6.87 ± 0.01	–9.76 ± 0.44
OH^–^	Δ*G*_9_	–205.93 ± 0.05	–197.74 ± 1.97
	Δ*G*_10_	–34.51 ± 0.08	–27.26 ± 2.21
	Δ*G*_11_	–120.16 ± 0.04	–113.82 ± 0.51
	Δ*G*_12_	–85.68 ± 0.04	–86.52 ± 0.09

a For OH^–^, we performed four thermodynamic cycles (eqs 9–12), resulting in free energies labeled as Δ*G*_9_ to Δ*G*_12_ (see text). All values are reported in kcal·mol^–1^.

It should be mentioned that, in addition to the mutual electrostatic and self-interaction energies, there is also a contribution for the liquid–vacuum interface^54^ (not included in Table 2). As explained in ref 54, this quantity is only dependent on the water model, which for TIP3P and SPCE­(E) amounts to −19 kcal·mol^–1^. Hence the values −120.22 and 85.68 kcal·mol^–1^ for HO^–^ and Na^+^ in bulk water become −101.22 and −104.68 kcal·mol^–1^, respectively, which now compare well with experimental values of −106.4 kcal·mol^–1^ ^55^ and −98.2 kcal·mol^–1^,^56^ respectively. Nonetheless, this contribution cancels out when the thermodynamic cycles are completed, but matching experimental values proves that the protocol is reliable.

The hydration free energy for transferring a water molecule from the bulk water to the reaction site of MAO A is therefore −2.9 kcal·mol^–1^ more favorable. For OH^–^, we obtained 6.3 kcal·mol^–1^ from eqs 9 and 10, and 7.7 kcal·mol^–1^ from eq 11, resulting in a total energy gap of 9.9 kcal·mol^–1^ (considering the average for OH^–^). Adding the free-energy value for hydroxide ion formation in bulk water by water protolysis of 23.88 kcal/mol, these results further support the conclusion that this reaction pathway concerning l-DOPA embedded in the MAO A protein is also not a relevant source of autoxidation.

## 4 Conclusions

The present study investigates the reaction mechanism of l-DOPA autoxidation incorporated in the enzyme monoamine oxidase A and replacing Tyr407. The proposed rate-limiting step is water protolysis in the vicinity of l-DOPA oxidized to its quinone form. The subsequent reactive step is an intramolecular Michael addition, which requires a hydroxide ion. This step has a substantially lower barrier and does not control the overall reaction kinetics. The simulation results clearly show that water protolysis, which is the rate-limiting step of l-DOPA autoxidation for the Tyr407L-DOPA MAO A mutant, has a barrier that is approximately 6 kcal mol^–1^ higher than that in bulk water. This provides substantial evidence that l-DOPA incorporated into MAO A, and presumably into other proteins as well, does not represent the predominant reaction channel for its autoxidation and therewith associated oxidative stress. Since l-DOPA autoxidation in water is a slow process with an experimental barrier of 27.55 kcal mol^–1^,^14^ it is plausible to assume that l-DOPA-induced oxidative stress may occur through a series of reactions, possibly following l-DOPA decarboxylation that results in dopamine, which can then autoxidize at a significantly faster rate.^10^

We demonstrated that the autoxidation reaction involving l-DOPA built in MAO A is a slow process. On the other hand, the fact that l-DOPA is indiscriminately incorporated into all central nervous system proteins, instead of aromatic amino acids, suggests that these altered proteins serve as a slow but consistent source of reactive oxygen species in Parkinson’s patients treated with l-DOPA. It is possible that certain regions of the protein, particularly in the vicinity of aromatic residues, may facilitate a catalytic environment favorable to water protolysis, and proteins containing zinc^57,58^ or calcium ions^59^ are among the primary candidates for such catalytic activity. In the presence of l-DOPA, these proteins could serve as a persistent source of oxidative stress. We also demonstrated that the pathway involving hydroxide ion formation in bulk water and the subsequent transport to the protein is not plausible. The experimental valence bond parameters that were derived in this study, together with the postulated reaction mechanism, could serve as a reference reaction for various protein environments. Comprehensive research efforts, including sequencing, structural analysis, kinetic studies, and clinical observations, are crucial to understand the side effects of l-DOPA and ideally to improve the pharmacological management of Parkinson’s disease. Our study represents an initial computational effort in this direction and lays the foundation for future investigations.

## Supplementary Material



## Notes

Free-energy profiles for various parametrizations provided in tables from S1 to S5; free-energy profile for the protolysis of a second water molecule which reaches the reaction site; details regarding the Lennard-Jones parametrization for hydrogen atoms of the reactive water molecules; and overview of the thermodynamic cycle used in alchemical transformations and a diagram for its steps in (PDF)

## References

[1] Rang, H. P. D. M. M. ; Ritter, J. M. ; Flower, R. J. Rand and Dale’s Pharmacology; Elsevier: Churchill Livingstone, 2007.

[2] Funk C. (1911). Synthesis
of dl-3:4-dihydroxyphenylalanine. J. Chem. Soc.,
Trans..

[3] Guggenheim M. (1913). Dioxyphenylalanin,
eine neue Aminosäure aus *Vicia faba*. Biol. Chem..

[4] Carlsson A., Lindqvist M., Magnusson T. O. R. (1957). 3,4-Dihydroxyphenylalanine
and 5-Hydroxytryptophan
as Reserpine Antagonists. Nature.

[5] Ehringer H., Hornykiewicz O. (1960). Verteilung von Noradrenalin und Dopamin (3-Hydroxytyramin)
im Gehirn Des Menschen und ihr Verhalten bei Erkrankungen des Extrapyramidalen
Systems. Klin. Wochenschr..

[6] Cotzias G. C., Van Woert M. H., Schiffer L. M. (1967). Aromatic amino acids and modification
of parkinsonism. N. Engl. J. Med..

[7] Yahr M. D., Duvoisin R. C., Schear M. J., Barrett R. E., Hoehn M. M. (1969). Treatment
of Parkinsonism with Levodopa. Arch. Neurol..

[8] Lees A. J., Tolosa E., Olanow C. W. (2015). Four pioneers
of L-dopa treatment:
Arvid Carlsson, Oleh Hornykiewicz, George Cotzias, and Melvin Yahr. Mov. Disord..

[9] Steele J. R., Strange N., Rodgers K. J., Padula M. P. (2021). A Novel Method for
Creating a Synthetic L-DOPA Proteome and In Vitro Evidence of Incorporation. Proteomes.

[10] Linert W., Herlinger E., Jameson R. F., Kienzl E., Jellinger K., Youdim M. B. H. (1996). Dopamine, 6-hydroxydopamine, iron, and dioxygen - Their
mutual interactions and possible implication in the development of
Parkinson’s disease. BBA, Mol. Basis
Dis..

[11] Almeida M. J., Machado J., Vieira Coelho M. A., Soares da Silva P., Coimbra J. (1998). l-3,4-Dihydroxyphenylalanine (l-DOPA)
secreted by oyster
(Crassostrea gigas) mantle cells: functional aspects. Comp. Biochem. Physiol., Part B:Biochem. Mol. Biol..

[12] Li Y., Cheng J., Delparastan P., Wang H., Sigg S. J., DeFrates K. G., Cao Y., Messersmith P. B. (2020). Molecular
design principles of Lysine-DOPA wet adhesion. Nat. Commun..

[13] Wonderly W. R., Cristiani T. R., Cunha K. C., Degen G. D., Shea J.-E., Waite J. H. (2020). Dueling
Backbones: Comparing Peptoid and Peptide Analogues
of a Mussel Adhesive Protein. Macromolecules.

[14] Prah A., Mavri J. (2024). L-DOPA Autoxidation:
An Empirical Valence Bond Simulation of the
Reactive Step. J. Phys. Chem. B.

[15] Åqvist J., Warshel A. (1992). Computer-Simulation
of the Initial Proton-Transfer
Step in Human Carbonic Anhydrase-I. J. Mol.
Biol..

[16] Warshel A., Weiss R. M. (1980). An Empirical Valence
Bond Approach for Comparing Reactions
in Solutions and in Enzymes. J. Am. Chem. Soc..

[17] Åqvist J. (1991). Free energy
perturbation study of metal ion-catalyzed proton transfer in water. J. Phys. Chem..

[18] Zhou Y. Z., Alany R. G., Chuang V., Wen J. (2012). Studies of the Rate
Constant of l-DOPA Oxidation and Decarboxylation by HPLC. Chromatographia.

[19] Neils T. L., Silverstein T. P., Schaertel S. (2023). Correction
to ″H(2)­O­(aq) Does
Not Exist: Critique of a Proof-of-Concept Derivation. J. Chem. Educ..

[20] Eigen M., De Maeyer L. (1955). Untersuchungen
über die Kinetik der Neutralisation.
I. Z. Elektrochem., Ber. Bunsenges. Phys. Chem..

[21] Lensink M. F., Mavri J., Berendsen H. J. C. (1999). Simulation
of slow reaction with
quantum character: Neutral hydrolysis of carboxylic ester. J. Comput. Chem..

[22] Son S. Y., Ma A., Kondou Y., Yoshimura M., Yamashita E., Tsukihara T. (2008). Structure
of human monoamine oxidase A at 2.2-angstrom
resolution: The control of opening the entry for substrates/inhibitors. Proc. Natl. Acad. Sci. U.S.A..

[23] Pettersen E. F., Goddard T. D., Huang C. C., Couch G. S., Greenblatt D. M., Meng E. C., Ferrin T. E. (2004). UCSF chimera - A
visualization system
for exploratory research and analysis. J. Comput.
Chem..

[24] Kaminski G.
A., Friesner R. A., Tirado-Rives J., Jorgensen W. L. (2001). Evaluation
and Reparametrization of the OPLS-AA Force Field for Proteins via
Comparison with Accurate Quantum Chemical Calculations on Peptides. J. Phys. Chem. B.

[25] Oanca G., Purg M., Mavri J., Shih J. C., Stare J. (2016). Insights into
enzyme point mutation effect by molecular simulation: phenylethylamine
oxidation catalyzed by monoamine oxidase A. Phys. Chem. Chem. Phys..

[26] Bayly C. I., Cieplak P., Cornell W., Kollman P. A. (1993). A well-behaved electrostatic
potential based method using charge restraints for deriving atomic
charges: the RESP model. J. Phys. Chem..

[27] Case D. A., Aktulga H. M., Belfon K., Cerutti D. S., Cisneros G. A., Cruzeiro V. W. D., Forouzesh N., Giese T. J., Götz A. W., Gohlke H. (2023). AmberTools. J. Chem. Inf. Model..

[28] Case, D. A. ; Cerutti, D. S. ; Cheatham, I. T. E. ; Darden, T. A. ; Duke, R. E. ; Giese, T. J. ; Gohlke, H. ; Goetz, A. W. ; Homeyer, N. Amber 2017; University of California: San Francisco: San Francisco, 2017.

[29] Frisch, M. J. ; Trucks, G. W. ; Schlegel, H. B. ; Scuseria, G. E. ; Robb, M. A. ; Cheeseman, J. R. ; Scalmani, G. ; Barone, V. ; Mennucci, B. ; Petersson, G. A. ; Gaussian 09; Gaussian, Inc.: Wallingford, CT, USA, 2009.

[30] Maestro; Schrödinger, LLC: New York, NY, 2025.

[31] Jorgensen W. L., Chandrasekhar J., Madura J. D., Impey R. W., Klein M. L. (1983). Comparison
of simple potential functions for simulating liquid water. J. Chem. Phys..

[32] Marelius J., Kolmodin K., Feierberg I., Åqvist J. Q. (1998). a molecular
dynamics program for free energy calculations and empirical valence
bond simulations in biomolecular systems. J.
Mol. Graphics Modell..

[33] Lee F. S., Warshel A. (1992). A Local Reaction Field Method for
Fast Evaluation of
Long-Range Electrostatic Interactions in Molecular Simulations. J. Chem. Phys..

[34] Lindahl E., Hess B., van der Spoel D. (2001). GROMACS 3.0: A Package for Molecular
Simulation and Trajectory Analysis. J. Mol.
Model..

[35] Bauer, P. ; Hess, B. ; Lindahl, E. GROMACS 2022.4 Source code, 2022. https://zenodo.org/records/7323393.

[36] Robertson M. J., Tirado-Rives J., Jorgensen W. L. (2015). Improved Peptide and Protein Torsional
Energetics with the OPLS-AA Force Field. J.
Chem. Theory Comput..

[37] Oanca G., van der Ent F., Åqvist J. (2023). Efficient
Empirical Valence Bond
Simulations with GROMACS. J. Chem. Theory Comput..

[38] Morgan B. R., Massi F. (2010). Accurate Estimates
of Free Energy Changes in Charge Mutations. J. Chem. Theory Comput..

[39] Berendsen, H. J. C. ; Postma, J. P. M. ; van Gunsteren, W. F. ; Hermans, J. Interaction Models for Water in Relation to Protein Hydration. In Intermolecular Forces; Pullman, B. , Ed.; D. Reidel: Dordrecht, 1981; Vol. 14, pp 331–342.

[40] Bennett C. H. (1976). Efficient
Estimation of Free-Energy Differences from Monte-Carlo Data. J. Comput. Phys..

[41] Hwang J. K., King G., Creighton S., Warshel A. (1988). Simulation of Free-Energy
Relationships and Dynamics of Sn2 Reactions in Aqueous-Solution. J. Am. Chem. Soc..

[42] Torrie G. M., Valleau J. P. (1977). Non-Physical Sampling Distributions in Monte-Carlo
Free-Energy Estimation - Umbrella Sampling. J. Comput. Phys..

[43] Zwanzig R. W. (1954). High-Temperature
Equation of State by a Perturbation Method 0.1. Nonpolar Gases. J. Chem. Phys..

[44] Torrie G. M., Valleau J. P. (1974). Monte-Carlo Free-Energy Estimates Using Non-Boltzmann
Sampling - Application to Subcritical Lennard-Jones Fluid. Chem. Phys. Lett..

[45] Prah A., Purg M., Stare J., Vianello R., Mavri J. (2020). How Monoamine
Oxidase A Decomposes Serotonin: An Empirical Valence Bond Simulation
of the Reactive Step. J. Phys. Chem. B.

[46] Warshel A. (1981). Calculations
of enzymic reactions: calculations of pKa, proton transfer reactions,
and general acid catalysis reactions in enzymes. Biochemistry.

[47] Warshel A., Sharma P. K., Kato M., Xiang Y., Liu H. B., Olsson M. H. M. (2006). Electrostatic Basis for Enzyme Catalysis. Chem. Rev..

[48] Singh N., Warshel A. (2010). A comprehensive examination of the contributions to
the binding entropy of protein-ligand complexes. Proteins.

[49] Born M. (1920). Volumes and
hydration warmth of ions. Z. Phys..

[50] Hünenberger P. H. (1999). Lattice-sum
methods for computing electrostatic interactions in molecular simulations. AIP Conf. Proc..

[51] Kastenholz M. A., Hünenberger P. H. (2006). Computation
of methodology-independent
ionic solvation free energies from molecular simulations. II. The
hydration free energy of the sodium cation. J. Chem. Phys..

[52] Hummer G., Pratt L. R., Garcia A. E. (1996). Free energy
of ionic hydration. J. Phys. Chem..

[53] Nijboer B. R. A., Ruijgrok T. W. (1988). On the Energy Per
Particle in Three-Dimensional and
Two-Dimensional Wigner Lattices. J. Stat. Phys..

[54] Åqvist J., Hansson T. (1998). Analysis of electrostatic
potential truncation schemes
in simulations of polar solvents. J. Phys. Chem.
B.

[55] Palascak M. W., Shields G. C. (2004). Accurate experimental
values for the free energies
of hydration of H+, OH-, and H3O+. J. Phys.
Chem. A.

[56] Burgess, M. A. Metal Ions in Solutions; Ellis Horwood: Chichester, England, 1978.

[57] Coleman J. E. (1992). Zinc Proteins
- Enzymes, Storage Proteins, Transcription Factors, and Replication
Proteins. Annu. Rev. Biochem..

[58] Frederickson C. J., Suh S. W., Silva D., Frederickson C. J., Thompson R. B. (2000). Importance of zinc in the central
nervous system: The
zinc-containing neuron. J. Nutr..

[59] Pidcock E., Moore G. R. (2001). Structural characteristics
of protein binding sites
for calcium and lanthanide ions. J. Biol. Inorg.
Chem..

