# Supplementary material for: l‑DOPA-Containing Protein Autoxidation: An Empirical Valence Bond Simulation of the Rate-Limiting Step
Source: J Phys Chem B. 2025 Nov 24;129(48):12422–31. doi: 10.1021/acs.jpcb.5c06223 (PMC12683634; doi:10.1021/acs.jpcb.5c06223)
Supplement: Supplementary file 1 [file jp5c06223_si_001.pdf]

Supporting information for:

L-DOPA-Containing Proteins Autoxidation: An Empirical Valence Bond Simulation of the Rate-Limiting Step

Gabriel Oanca[1], Alja Prah[2,3], Johan Åqvist[1], Janez Mavri[2*]

1 Department of Cell & Molecular Biology, Uppsala University, Biomedical Center, SE-751 24 Uppsala, Sweden

2 Laboratory for Computational Biochemistry and Drug Design, National Institute of Chemistry, Hajdrihova 19, 1000 Ljubljana, Slovenia

3 Networking Infrastructure Centre, Jožef Stefan Institute, Jamova 39, 1000 Ljubljana, Slovenia

[*] Corresponding author: janez.mavri@ki.si

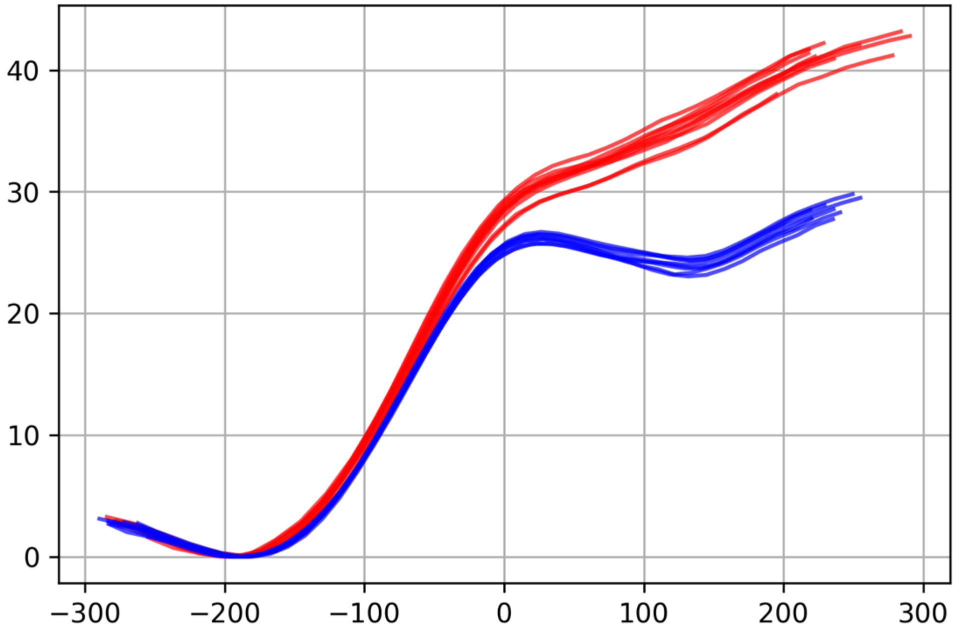

Figure S1: Reaction profiles for water protolysis using the parameters from Table S1.

Table S1: Parameters pertaining to profiles in Figure S1. Lennard-Jones parameters are provided in Q force-field format, *i.e.*, as the square root of their standard form due to the use of geometric combination rule. All values are in AKMA units.

Atom types	Atomic Charges[1]	Lennard-Jones[2]		Buckingham repulsion[3]	
		A	B	C	β
O (H_2O)	-0.834000	762.8900	24.3900	10.0	1.58
H (H_2O)	0.417000	59.8020	3.8273	50.0	1.58
O (H_3O^+)	-0.755000	762.8900	24.3900	--	--
H (H_3O^+)	0.585000	59.8020	3.8273	50.0	1.58
O (OH^-)	-1.205424	976.9297	31.2559	30.0	1.58
H (OH^-)	0.205424	69.5797	4.9095	--	--

Morse bonds[4]			
Atom pairs	D_e	β	r_0
O – H (H_2O)	138.5	2.0	1.0
O – H (OH^-)	332.5	2.0	1.0
O – H (H_3O^+)	100.0	2.0	1.0

EVB parameters		Energies	Water	Enzyme
H_{ij}	48.50	$\Delta G^‡$	26.21 ± 0.33	--
gas-shift	284.23	ΔG_0	23.87 ± 0.52	--

1. TIP3P (H_2O) and ffld (OH^- and H_3O^+) charges.
2. TIP3P (H_2O) and ffld (OH^- and H_3O^+) Lennard-Jones parameters for oxygens and hydroxide H atom. For the other H atoms, we used the same parameters as for H28 of lumiflavin (ffld_server generated[1]).
3. Buckingham type potential has only been applied to pairs in breaking and forming bonds.
4. Morse D_e parameters were calculated as: $D_e = k/(2 \cdot \beta^2)$, where *k* is the harmonic constant of TIP3P model for H_2O and ffld_server generated for H_3O^+ and OH^-. Morse potential was applied to all EVB bonds.

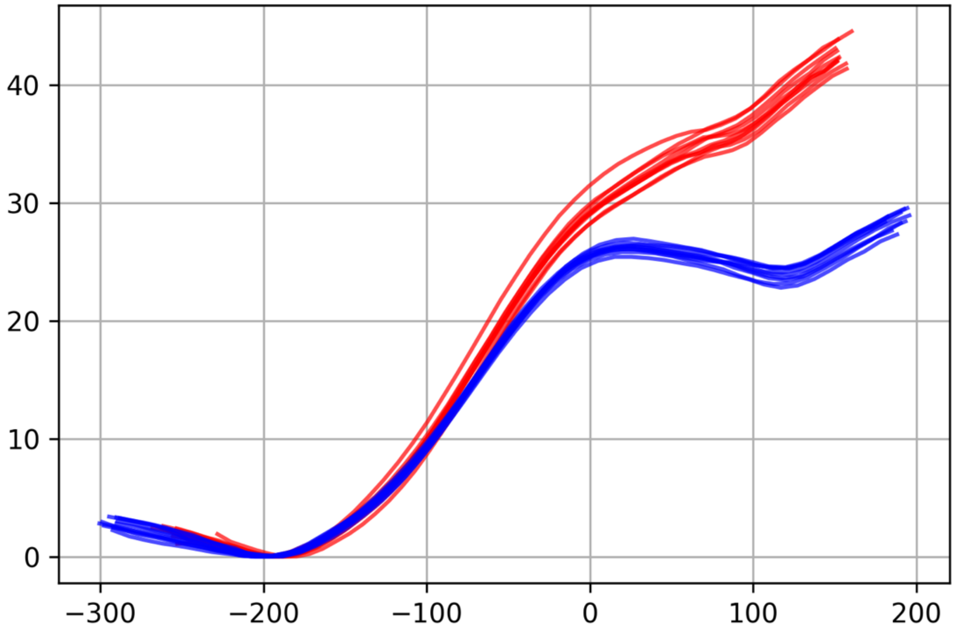

Figure S2: Reaction profiles for water protolysis using the parameters from Table S2.

Table S2: Parameters pertaining to profiles in Figure S2. Lennard-Jones parameters are provided in Q force-field format, *i.e.*, as the square root of their standard form due to the use of geometric combination rule. All values are in AKMA units.

Atom types	Atomic Charges[1]	Lennard-Jones[2]		Buckingham repulsion[3]	
		A	B	C	β
O (H_2O)	-0.80	762.8900	24.3900	15.0	1.58
H (H_2O)	0.40	6.2400	1.4700	50.0	1.58
O (H_3O^+)	-0.08	762.8900	24.3900	--	--
H (H_3O^+)	0.36	6.2400	1.4700	50.0	1.58
O (OH^-)	-1.01	976.9297	31.2559	15.0	1.58
H (OH^-)	0.01	6.3100	1.4800	--	--

Morse Bonds[4]			
Atom pairs	D_e	β	r_0
O-H	109.1	2.0	1.0

EVB parameters		Energies	Water	Enzyme
H_{ij}	49.00	$\Delta G^‡$	26.21 ± 0.40	--
gas-shift	207.06	ΔG_0	23.88 ± 0.59	--

1. Atomic charges taken from reference [2].
2. TIP3P (H_2O) and ffld (OH^- and H_3O^+) Lennard-Jones parameters for oxygen atoms. Parameters for hydrogen atoms were calculated as explained below.
3. Buckingham type potential has only been applied to pairs in breaking and forming bonds.
4. Morse potentials were applied to all EVB bonds.

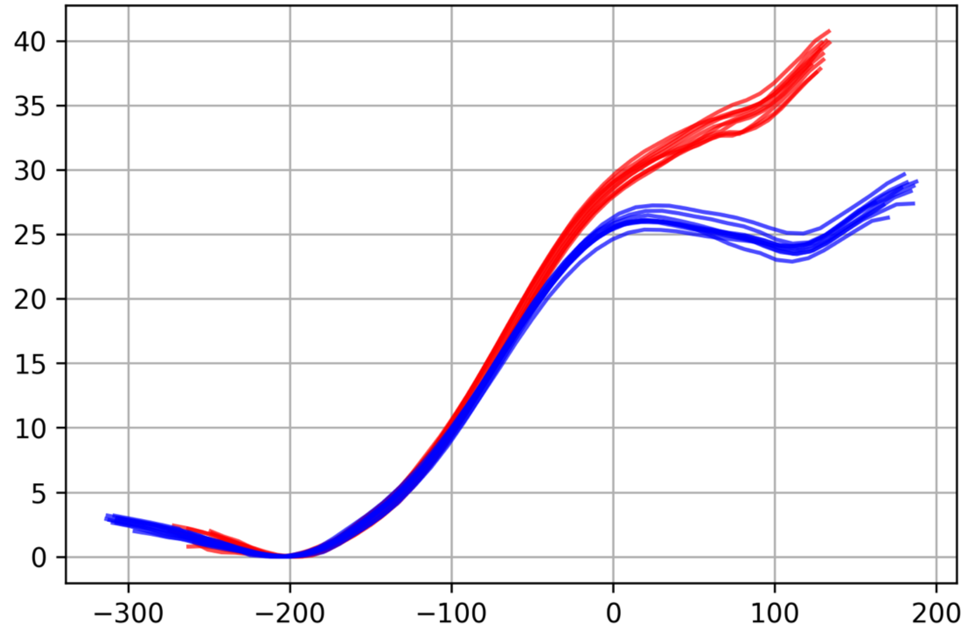

Figure S3: Reaction profiles for water protolysis using the parameters from Table S3.

Table S3: Parameters pertaining to profiles in Figure S3. Lennard-Jones parameters are provided in Q force-field format, *i.e.*, as the square root of their standard form due to the use of geometric combination rule. All values are in AKMA units.

Atom types	Atomic Charges[1]	Lennard-Jones[2]		Buckingham repulsion[3]	
		A	B	C	β
O (H_2O)	-0.80	762.8900	24.3900	15.0	1.58
H (H_2O)	0.40	6.2400	1.4700	50.0	1.58
O (H_3O^+)	-0.08	762.8900	24.3900	--	--
H (H_3O^+)	0.36	6.2400	1.4700	50.0	1.58
O (OH^-)	-1.01	976.9297	31.2559	15.0	1.58
H (OH^-)	0.01	69.5797	4.9095	--	--

Morse bonds[4]			
Atom pairs	D_e	β	r_0
O-H	109.1	2.0	1.0

EVB parameters		Energies	Water	Enzyme
H_{ij}	49.10	$\Delta G^‡$	26.21 ± 0.49	--
gas-shift	205.75	ΔG_0	23.88 ± 0.55	--

1. Atomic charges taken from reference [2].
2. TIP3P (H_2O) and ffld (OH^- and H_3O^+) Lennard-Jones parameters for oxygen atoms. Parameters for hydrogen atoms were calculated as explained below.
3. Buckingham type potential has only been applied to atom pairs in breaking and forming bonds.
4. Morse potentials were applied to all EVB bonds.

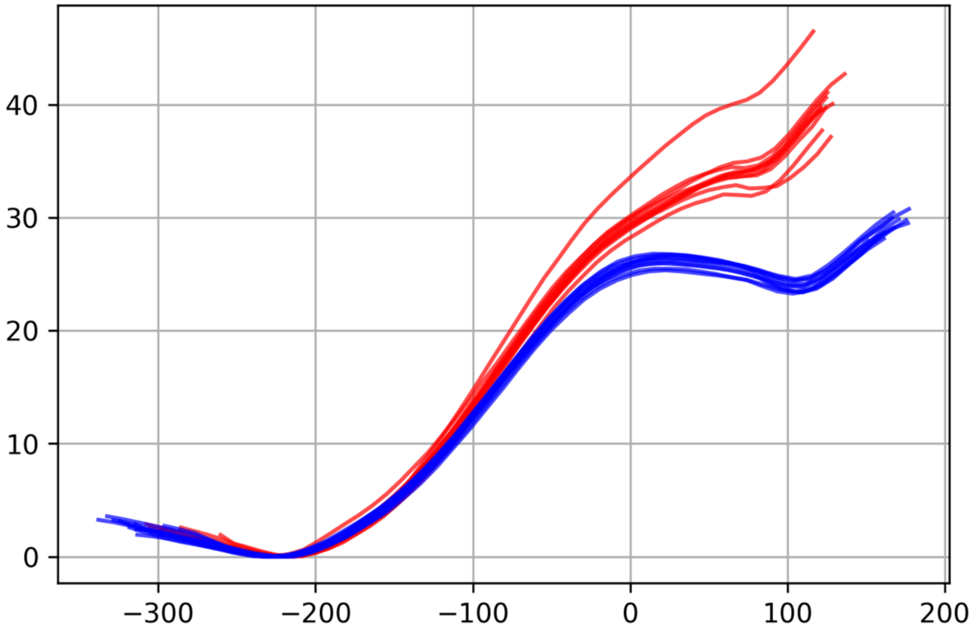

Figure S4: Reaction profiles for water protolysis using the parameters from Table S4.

Table S4: Parameters pertaining to profiles in Figure S4. Lennard-Jones parameters are provided in Q force-field format, *i.e.,* as the square root of their standard form due to the use of geometric combination rule. All values are in AKMA units.

Atom types	Atomic Charges[1]	Lennard-Jones[2]		Buckingham repulsion[3]	
		A	B	C	β
O (H_2O)	-0.80	762.8900	24.3900	15.0	1.58
H (H_2O)	0.40	6.2400	1.4700	50.0	1.58
O (H_3O^+)	-0.08	762.8900	24.3900	--	--
H (H_3O^+)	0.36	6.2400	1.4700	50.0	1.58
O (OH^-)	-1.01	976.9297	31.2559	20.0	1.58
H (OH^-)	0.01	69.5797	4.9095	--	--

Morse bonds[4]			
Atom pairs	D_e	β	r_0
O-H	109.1	2.0	1.0

EVB parameters		Energies	Water	Enzyme
H_{ij}	56.38	$\Delta G^‡$	26.21 ± 0.49	--
gas-shift	209.12	ΔG_0	23.88 ± 0.47	--

1. Atomic charges taken from reference [2]
2. TIP3P (H_2O) and ffld (OH^- and H_3O^+) Lennard-Jones parameters for oxygen atoms. Parameters for hydrogen atoms were calculated as explained below.
3. Buckingham type potential has only been applied to atom pairs in breaking and forming bonds.
4. Morse potentials were applied to all EVB bonds.

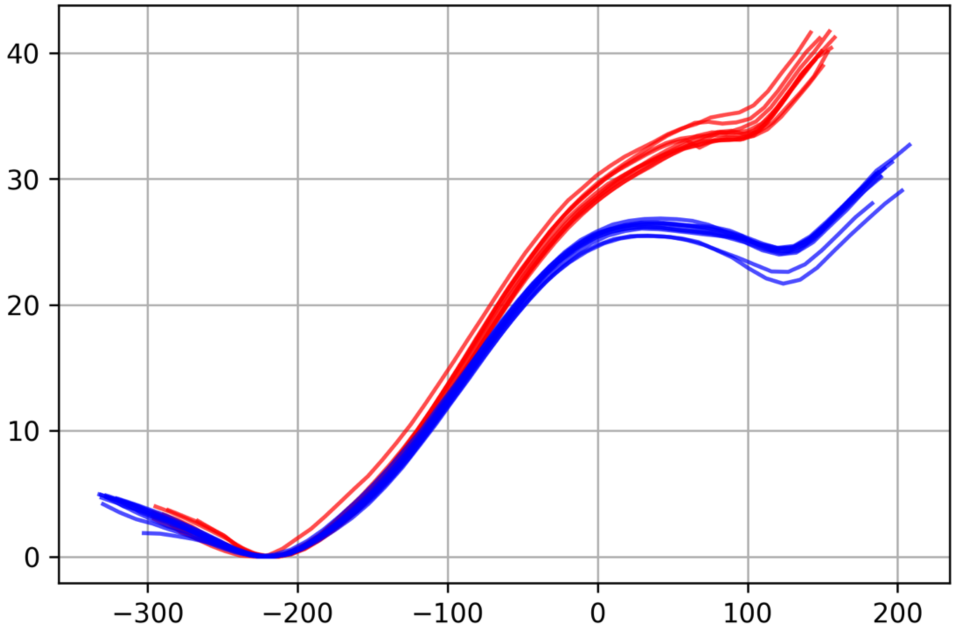

Figure S5: Reaction profiles for water protolysis using the parameters from Table S5.

Table S5: Parameters pertaining to profiles in Figure S5. Lennard-Jones parameters are provided in Q force-field format, *i.e.,* as the square root of their standard form due to the use of geometric combination rule. All values are in AKMA units.

Atom types	Atomic Charges[1]	Lennard-Jones[2]		Buckingham repulsion[3]	
		A	B	C	β
O (H_2O)	-0.80	762.8900	24.3900	20.0	1.58
H (H_2O)	0.40	6.2400	1.4700	50.0	1.58
O (H_3O^+)	-0.08	762.8900	24.3900	--	--
H (H_3O^+)	0.36	6.2400	1.4700	50.0	1.58
O (OH^-)	-1.01	976.9297	31.2559	20.0	1.58
H (OH^-)	0.01	69.5797	4.9095	--	--

Morse bonds[4]			
Atom pairs	D_e	β	r_0
O-H	109.1	2.0	1.0

EVB parameters		Energies	Water	Enzyme
H_{ij}	67.08	$\Delta G^‡$	26.21 ± 0.42	
gas-shift	209.09	ΔG_0	23.88 ± 0.91	

1. Atomic charges taken from reference [2]
2. TIP3P (H_2O) and ffld (OH^- and H3O+) Lennard-Jones parameters for oxygen atoms. Parameters for hydrogen atoms were calculated as explained below.
3. Buckingham type potential has only been applied to atom pairs in breaking and forming bonds.
4. Morse potentials were applied to all EVB bonds.

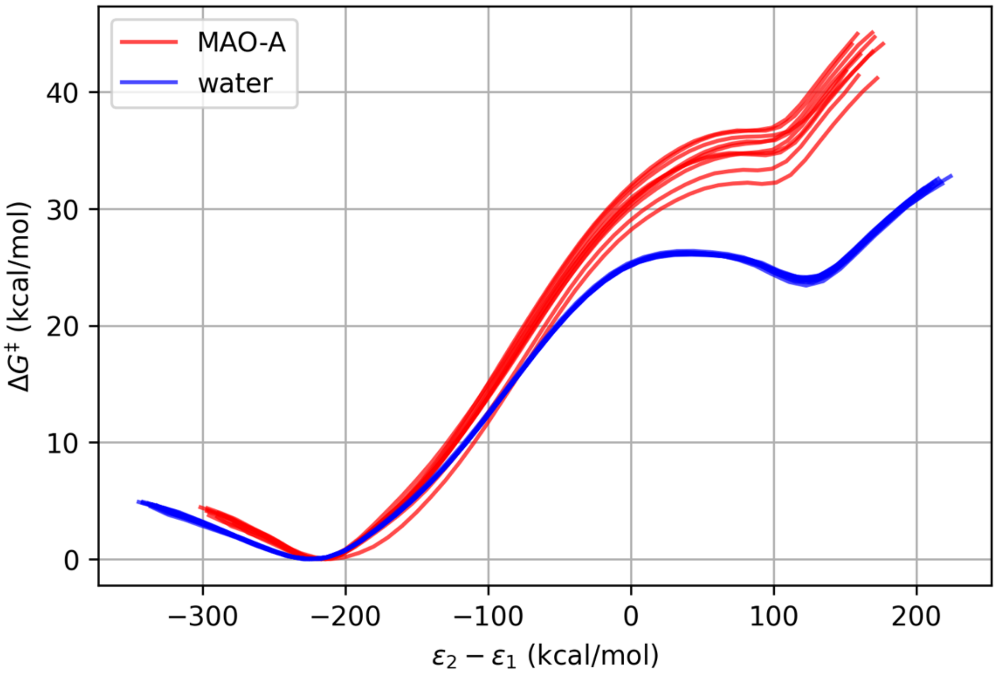

Figure S6: Reaction profiles for water protolysis, where the proton acceptor is the molecule indicated in blue in Figure 3 in the main text. For these simulations, we used the set of parameters from Table 1 in the main text.

Lennard-Jones parameters for hydrogen atoms

The Lennard-Jones parameters (LJ) for OH^- and H_3O^+ hydrogen atoms in Tables S2 to S5 in this file and Table 1 in the main text were generated as follows:

For any two atoms with LJ parameters σ_i and ε_i, the van der Waals potential shows a minimum at the distance $r_{ij} = 1.122\sqrt{\sigma_i \sigma_i}$. TIP3P water model does not generate van der Waals interactions for hydrogen atoms (σ_i and ε_i equal zero). In our case, we assigned to the hydrogen atoms of the reactive water molecules such parameters that its potential minimum, when interacting with the oxygen atoms from the first solation shell, will overlap with the minimum between the two oxygen atoms. The LJ equilibrium distance between two TIP3P water molecules is 3.534 Å, and the O-H Morse equilibrium distance is 1 Å (see Table 1 in the main text).

$$\sigma_h = \left(\frac{r_{oo} - 1}{1.122}\right)^2 \Big/ \sigma_o$$

Then we used the value ε from the alpha hydrogen atoms (HA type in OPLS-AA force field) to calculate *A* and *B* parameters: $A_h=\sqrt{4\varepsilon_h\sigma_h{}^{12}}$, and $B_h=\sqrt{4\varepsilon_h\sigma_h{}^{6}}$

The Lennard-Jones potential between two atoms *i* and *j* is $V_{ij}=\frac{A_{ij}}{r^{12}}-\frac{B_{ij}}{r^{6}}$, where $A_{ij}=\sqrt{A_iA_j}$ and $B_{ij}=\sqrt{B_iB_j}$. We want to note that in the force field libraries of Q software, these values are already provided as square roots, to speed up calculations, which is also how we have presented them in all the tables in this file and in the main text.

Thermodynamic Cycle: Overview

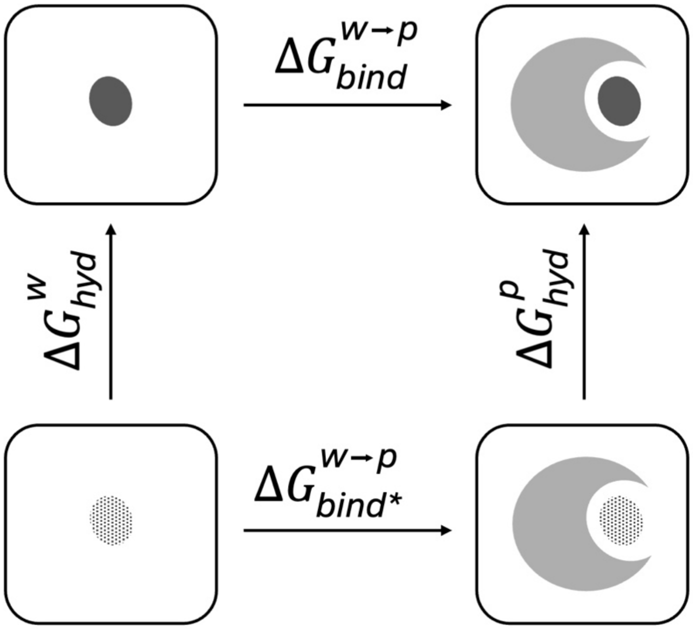

Figure S7: Scheme of a typical thermodynamic cycle used for calculating the free energy of ligand binding. The small spere represents the ligand, while the semicircle on the right column represents the protein. Dark colored ligand on the upper row indicates being in the charged state, while the shaded ligand on the bottom row indicates being in the uncharged state. Hydration free energy in water and protein environment is labeled as ΔG_{hyd}^{w} and ΔG_{hyd}^{p}, respectively. Binding free energy is labeled as $\Delta G_{bind}^{w\rightarrow p}$, while $\Delta G_{bind*}^{w\rightarrow p}$ indicates the free energy of binding an uncharged ligand.

Thermodynamic cycle (TC) is the standard method for calculating the binding free energy of a ligand to a protein. The diagram from Figure S7 shows such a TC in its simplest form, but precise

quantitative calculations usually require several TCs. The binding free energy represents the free energy required to transfer the ligand from water to the protein environment:

$$\Delta G_{bind}^{w \to p} = G^p - G^w$$

where G^p and G^w is the solvation free energy in protein and water, respectively. Such calculations are not trivial if we simulate the transformation along geometrical coordinates. A computationally more efficient approach is to calculate the solvation free energy in water and in protein, separately, using free energy perturbations (FEP). In this method, the ligand is mutated from an uncharged state, where it does not interact with the environment, to a charged state, a technique known as "alchemical transformation". In this scenario, we obtain the following equality:

$$\Delta G_{bind}^{w \to p} = -\Delta G_{hyd}^{w} + \Delta G_{bind^*}^{w \to p} + \Delta G_{hyd}^{p}$$

For accurate quantitative calculations, one must mutate the ligand from being complete dummy (*i.e.*, no electrostatics nor van der Waals interactions) to being fully charged. For a dummy substrate, $\Delta G_{bind^*}^{w \to p}$ equals zero and only the left and right legs of the thermodynamics cycle will contribute to the binding free energy:

$$\Delta G_{bind}^{w \to p} = \Delta G_{hyd}^{p} - \Delta G_{hyd}^{w}$$

In this work, we have only mutated the electrostatic charges and have ignored the differences in the van der Waals contribution between H_2O and OH^- in water and in the enzyme. In our case, overlooking this small contribution would not have altered the conclusion that water deprotonation does not occur at the reaction site of the MAO A protein.

References:

1. Oanca, G.; Purg, M.; Mavri, J.; Shih, J. C.; Stare, J., Insights into enzyme point mutation effect by molecular simulation: phenylethylamine oxidation catalyzed by monoamine oxidase A. *Phys Chem Chem Phys* **2016,** *18* (19), 13346-13356.

2. Åqvist, J., Free energy perturbation study of metal ion-catalyzed proton transfer in water. *The Journal of Physical Chemistry* **1991,** *95* (12), 4587-4590.
